# 

**Published:** 1897-03-27

**Authors:** 


					THE HOSPITAL. ABSOLUTELY PURE, therefore BEST. March 27th, 1897."
CADBURY'S mr CADBURY'S
COCOA
' The standard of highest purity at present ML <2?* Mn atvattpc riccr*
attainable."?Art m/v/1. ^ i?NO ALKALIhS USED
COCOA
?7
isaonr Western luFi/tutnr
'flff/CH HOSPIJAL "
WUU/T W1 tiH l IlJvN t Nr lafmAaJ
No. .548 Vol. XXI. |JK?p?l Satubday,
March 27th, 1897,
E Subscription Terms,
see p. 426.
] PRICE TWOPENCE.

				

